# Mr. Sutliffe on Antimony and Calomel in Typhus

**Published:** 1813-10

**Authors:** Edward Sutliffe

**Affiliations:** Bread-street Hill


*To the Editors of the Medical and Physical Journal.*

GENTLEMEN,

THE following case having come recently under my care, I have taken the liberty to transmit you the particulars, not so much as possessing peculiar claims to your attention, as to prove the decided efficacy of medicine.

 —— Ellis,

—— Ellis, a young woman of 20 years of age, I was called to see in my vicinity laboring under typhus, apparently of a peculiar malignity. Immediately on entering the room I complained of the excessive fœtor, and inquired as to the state of the bowels; and was informed that every thing passed away involuntarily. Her pulse was feeble and quick. Her complaint was constantly of the head, and very significantly putting her hands to the sides of her head. Her tongue (shown by tremulous attempts) was covered with brown fur, and approaching sordes on the teeth and gums. Her anxiety and restlessness was such that she would have picked off every species of clothing but for surrounding attendants; and a constant muttering delirium, with occasional paroxysms of violence. Her incapacity for conversation, or to hearken to any thing that could have been suggested to her, rendered her situation truly deplorable.

I advised that she should have infusion of tea liberally; opened the window, and desired it to remain so; put on a large blister upon the nape and shoulders; and gave Pulv. Antim. gr. xij. and Calomel, gr. vj. to be taken in honey every twelve hours. The first dose caused a profuse perspiration; the second acted usefully upon the bowels. She took four more such doses, and the feculent matter assumed a less diseased appearance gradually. At this period the head-ache had entirely left her; the motions were voluntary and relieving; the pulse firm and 86; the brown fur substituted by a greyish hue, but that moist, and confined to the centre of the tongue; looks distinctly at me, and smiles with gratitude; wants pen and ink to write to her father in Yorkshire. The day following I called to see that she had not relapsed, and found she had slept quite sound, and eaten several oysters.

I think I may affirm, without the fear of contradiction, that had this young subject been loaded with bark and opium, she would soon have ceased to exist; and allow me to acknowledge my small tribute of the incalculable obligations medical men in particular, and society in general, are laid under to the laborious exertions and judicious treatment recommended by Dr. Clutterbuck in such fevers.

I have the honor to subscribe myself,

Your's, &c.

EDWARD SUTLIFFE.

*Bread-street Hill,*

*Sept.* 8, 1813.

COLLEC-

